# Identification and validation of stable reference genes for RT-qPCR analysis in blood samples from golden Syrian hamsters under different immunological conditions

**DOI:** 10.1007/s11033-026-12980-8

**Published:** 2026-10-23

**Authors:** Jady Duarte Nogueira, Tiffany Thurow Bunde, Mara Andrade Colares Maia, Natasha Rodrigues de Oliveira, Odir Antônio Dellagostin, Thaís Larré Oliveira Bohn

**Affiliations:** 1 Laboratório de Vacinologia, Centro de Desenvolvimento Tecnológico, Universidade Federal de Pelotas, Pelotas, Rio Grande do Sul Brasil; 2 Instituto de Ciências Biológicas, Universidade Federal do Rio Grande, Rio Grande, Rio Grande do Sul Brazil

**Keywords:** Gene expression normalization, Leptospirosis, RefSeeker, *ppia*

## Abstract

**Background:**

The golden Syrian hamster is the main experimental model for leptospirosis. However, there are no validated reference genes for reverse transcription quantitative PCR (RT-qPCR) analysis of blood samples.

**Methods and Results:**

The expression stability of 11 candidate reference genes was evaluated in hamster blood under distinct immunological conditions, including *Leptospira interrogans* infection and different immunization strategies. Stability was assessed using BestKeeper, geNorm, NormFinder, and the comparative ΔCt method, with the consensus ranking calculated using the RefFinder approach implemented in the RefSeeker R package. The most stable reference gene was *ppia*, followed by *gapdh* and *ppib*, whereas *actb* was the least stable gene in the ranking. Normalization of *tnf-α* expression using *actb* resulted in an approximately 10-fold overestimation compared with *ppia*, demonstrating the impact of reference gene choice on data interpretation.

**Conclusions:**

These findings support *ppia* as a reliable reference gene for RT-qPCR normalization in hamster blood under both infection and vaccination conditions.

**Supplementary Information:**

The online version contains supplementary material available at https://doi.org/10.1007/s11033-026-12980-8.

## Introduction

*Leptospira interrogans* is a pathogenic spirochete responsible for leptospirosis, a globally distributed zoonotic disease of major public health concern [1]. Considerable efforts have been devoted to understanding host–pathogen interactions and developing effective vaccines against leptospirosis [1]. The golden Syrian hamster (*Mesocricetus auratus*) is highly susceptible to acute *Leptospira* infection and is therefore widely used as a major experimental model for this disease [2, 3]. However, the limited availability of species-specific reagents, particularly for protein-based immunological analyses, remains a major limitation of this model. Consequently, reverse transcription quantitative PCR (RT-qPCR) has become an important tool for assessing host immune responses at the transcriptional level [4, 5]. Among the available biological matrices, blood is particularly valuable because it provides insight into systemic immune responses and enables less invasive sampling strategies [6].

Building on our previous work, in which suitable reference genes were identified for kidney, liver, and lung tissues of hamsters under vaccination and infection conditions [7], the present study focuses on blood as a complementary and highly informative matrix. Blood sampling offers practical advantages due to its accessibility and potential application in longitudinal experimental designs, while also providing information on systemic immune responses relevant to infection progression and vaccine evaluation. However, because blood is highly responsive to physiological and immunological changes, accurate RT-qPCR analysis in this matrix requires rigorous normalization using stable internal reference genes [8].

The selection of an appropriate reference gene, or endogenous control, is one of the most critical steps in RT-qPCR experimental design. A suitable reference gene should exhibit stable basal expression across samples and remain unaffected by experimental treatments or conditions [9]. Nevertheless, commonly used genes such as *gapdh* and *actb* are often selected based on convention rather than prior validation within the specific biological matrix and experimental context under investigation [8]. Because reference gene stability is tissue- and condition-dependent, experimental validation is strongly recommended for each model, sample type, and treatment protocol.

Given the relevance of blood for investigating systemic host responses during leptospirosis and vaccine evaluation, and considering the lack of validated reference genes for RT-qPCR analysis in this matrix, the present study aimed to identify stable reference genes in blood samples from golden Syrian hamsters under diverse immunological conditions. These conditions included *L. interrogans* infection and immunization using distinct formulations, including bacterin, aluminum hydroxide, *Mycobacterium bovis* BCG, and attenuated *Salmonella enterica* serovar Typhimurium. These strategies were selected to encompass diverse vaccine platforms, adjuvants, and delivery systems relevant to experimental vaccine development, as well as their use in studies involving *Leptospira* spp., SARS-CoV-2, infectious bronchitis virus, and respiratory syncytial virus [3, 10–12]. By validating candidate reference genes across these conditions, this study provides a methodological basis for more accurate and reproducible RT-qPCR analyses in hamster blood.

## Materials and methods

### Biological samples and animal conditions

Blood samples used in this study were obtained from a previous experiment conducted by our group, as described elsewhere [7]. Briefly, 4–6-week-old male and female golden Syrian hamsters (*Mesocricetus auratus*) were randomly assigned to six experimental groups (*n* = 4 per group). One group was infected with *Leptospira interrogans* serovar Copenhageni strain Fiocruz L1-130 (~ 1.5 × 10⁴ cells/mL) and monitored until predefined clinical endpoints were reached, including hematuria, nasal bleeding, anorexia, impaired locomotion or breathing, ruffled fur, hunched posture, prostration, or body weight loss exceeding 10%. The remaining groups received two-dose immunization protocols with ovalbumin adsorbed to 2% aluminum hydroxide, attenuated *Salmonella enterica* serovar Typhimurium (2 × 10⁷ CFU), inactivated *L. interrogans* bacterin (10⁸ CFU) at 14-day intervals. Animals in the PBS control group received two doses of PBS following the same immunization schedule as the vaccinated groups and served as a mock-immunized negative control. Animals immunized with *Mycobacterium bovis* BCG Pasteur (10⁶ CFU) received two doses at a 21-day interval. Euthanasia was performed at the clinical endpoint for infected animals or 21 days after the booster dose for vaccinated animals. Blood samples were aseptically collected by cardiac puncture, immediately mixed with RNAlater stabilization solution, and stored at − 80 °C until processing. All procedures were approved by the Animal Ethics Committee of the Federal University of Pelotas (UFPel), Brazil, under protocol CEUA 022906/2023-38.

### Total RNA extraction and cDNA synthesis

Total RNA was extracted from whole blood using a modified protocol combining TRIzol™ Reagent (Invitrogen, USA) and the RNeasy^®^ Mini Kit (Qiagen, USA). Briefly, concentrated blood pellets were resuspended in 500 µL of RNase-free water, lysed with TRIzol™, and subjected to chloroform phase separation by centrifugation at 12,000 × *g* for 15 min at 4 °C. The aqueous phase was recovered, mixed with an equal volume of absolute ethanol, and applied directly to RNeasy spin columns. After column washing, RNA was eluted twice with 35 µL of pre-warmed RNase-free water at 60 °C to maximize yield. RNA concentration and purity were determined using a NanoDrop Lite Spectrophotometer (Thermo Fisher Scientific, USA). For cDNA synthesis, 1,000 ng of total RNA, with A260/280 ratios ranging from 1.92 to 2.08, was reverse-transcribed using the High-Capacity cDNA Reverse Transcription Kit (Applied Biosystems, USA), which uses random primers, in a final volume of 40 µL, according to the manufacturer’s instructions.

### Candidate reference genes and primer design

The 11 candidate reference genes evaluated in this study were selected from our previous work [7]. This panel included three categories: four novel candidates identified by RNA-seq analysis in hamsters (*rps16*, *aprt*, *gpx1*, and *fkbp1a*), four non-conventional reference genes (*ppia*, *ppib*, *pgk1*, and *rps29*), and three classical reference genes commonly used for RT-qPCR normalization (*ywhaz*, *actb*, and *gapdh*). Primers were designed using Primer3Plus according to MIQE guidelines [8]. The complete list of primer sequences and their respective amplification efficiencies are available in the Supplementary Material (Supplementary Table S1). Amplification specificity was confirmed by the presence of a single band on agarose gel electrophoresis and a single peak in melting curve analysis (Supplementary Figure S2).

### Real-time PCR analysis

Real-time PCR amplification was performed using the QuantStudio™ 3 Real-Time PCR System (Applied Biosystems, USA). Reactions were prepared in a final volume of 12.5 µL containing 100 ng of cDNA, 6.25 µL of SYBR Green Master Mix (Promega, USA), and 0.1–0.5 µM of each primer. All reactions were performed in duplicate. The cycling conditions consisted of an initial denaturation at 95 °C for 60 s, followed by 40 cycles of denaturation at 95 °C for 10 s and annealing/extension at either 60–62 °C for 30 s, according to the primer pair used. No-template controls were included in each run to monitor potential reagent contamination or nonspecific amplification. After amplification, melting curve analysis was performed to verify product specificity using a temperature ramp from 60 °C to 95 °C, with increments of 4.4 °C per step.

### Statistical analysis

Reference gene stability was analyzed using R statistical software version 4.5.1 (R Foundation for Statistical Computing, Austria) within the RStudio environment (RStudio, USA). The RefSeeker R package [13] was used to implement an integrated workflow based on four widely used algorithms for reference gene stability analysis: geNorm [14], NormFinder [15], BestKeeper [16], and the comparative ΔCt method [17]. These algorithms assess expression stability using distinct statistical approaches: BestKeeper calculates the mean absolute deviation of raw Ct values, geNorm estimates the average pairwise variation or M value, NormFinder evaluates intra- and inter-group expression variance, and the comparative ΔCt method determines the standard deviation of ΔCt values across gene pairs. Furthermore, geNorm was used to perform pairwise variation analysis (V_n/*n*+1)_ between sequential normalization factors to determine the optimal number of reference genes required for accurate normalization, adopting the conventional threshold of 0.15. The final consensus ranking was calculated using the RefFinder approach [18], implemented through the RefSeeker R package [13]. For each candidate gene, the geometric mean of the ranks assigned by BestKeeper, geNorm, NormFinder, and the comparative ΔCt method was calculated, with lower geometric mean values indicating greater overall stability. This approach integrates differences among the individual algorithms into a single consensus ranking. RefSeeker was also used to generate formatted tables, spreadsheets, and graphical outputs for data visualization.

### Impact of reference gene selection on target gene expression

The expression of *tnf-α* was analyzed to assess the impact of reference gene selection on target gene quantification. For this analysis, the most stable and least stable reference genes, as determined by the overall RefSeeker ranking, were used to normalize *tnf-α* expression in blood samples from hamsters infected with *L. interrogans*, while samples from PBS control group were used as calibrators for the ΔΔCt calculation. Considering the recommendation of pairwise variation analysis, the normalization strategy also included the mean of the three most stable genes. The *tnf-α* gene and the infection condition were selected based on previous evidence showing increased *tnf-α* expression in infected animals [19]. *tnf-α* is a key pro-inflammatory cytokine involved in systemic inflammation, making its expression in blood a relevant target for evaluating host responses to *Leptospira* infection. PCR amplification was performed under the same conditions described above. Relative expression levels were calculated using the comparative ΔΔCt method. Statistical differences were assessed by one-way analysis of variance (ANOVA), with significance defined as *p* < 0.05. Analyses were performed using GraphPad Prism version 8.

## Results and discussion

### Evaluation of candidate reference gene expression stability

#### Comprehensive ranking of reference genes

Accurate normalization is essential for the reliable interpretation of RT-qPCR gene expression data. In this study, we evaluated the stability of 11 candidate reference genes in blood samples from golden Syrian hamsters under vaccination and infection conditions. The selected panel included classical reference genes commonly used in RT-qPCR studies (*actb*, *gapdh*, and *ywhaz*), non-conventional candidates previously reported as stable in different tissues and animal models (*pgk1*, *ppia*, *ppib*, and *rps29*), and novel candidates identified by RNA-seq or previously validated in hamsters (*aprt*, *fkbp1a*, *gpx1*, and *rps16*) [20]. Gene stability was assessed both within each experimental condition and across all samples to identify condition-specific and overall reference gene performance.

**Fig. 1:**
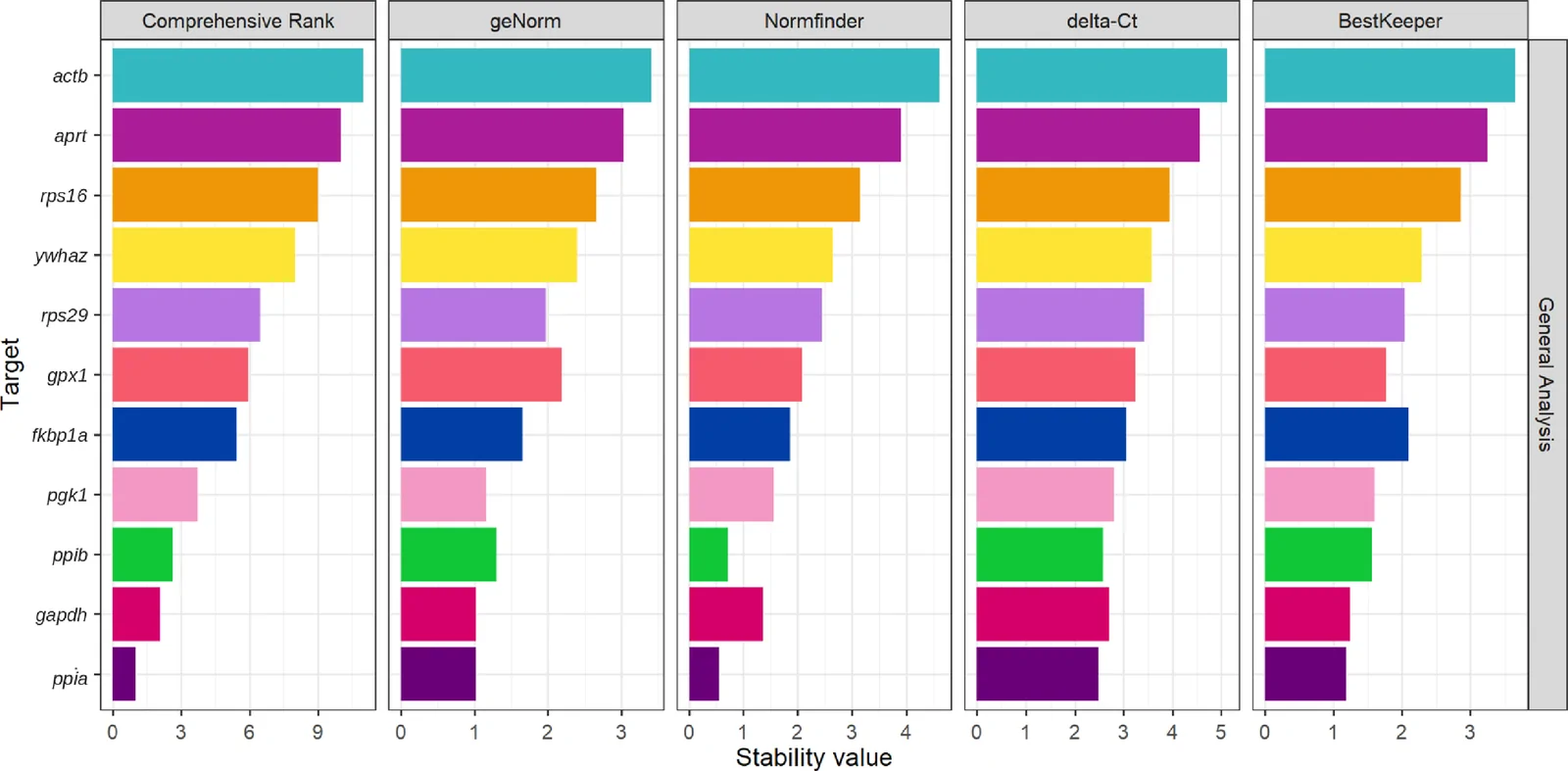
Overall stability ranking of candidate reference genes across all blood samples and experimental conditions. Gene stability was assessed using BestKeeper, geNorm, NormFinder, and the comparative ΔCt method. The final consensus ranking was calculated using the RefFinder approach implemented in the RefSeeker R package. Lower ranking values indicate greater expression stability

To establish a consensus ranking, the RefFinder approach [18] was implemented using the RefSeeker R package [13]. This workflow integrates the rankings generated by BestKeeper, geNorm, NormFinder, and the comparative ΔCt method, and calculates a final consensus ranking based on the geometric mean of the individual rankings. The 11 candidate genes were evaluated across six experimental conditions: immunization with ovalbumin formulated with aluminum hydroxide, BCG Pasteur, attenuated *S. Typhimurium*, or inactivated *L. interrogans* bacterin, as well as PBS administration (control group) and active *L. interrogans* infection. Across all samples, *ppia* was identified as the most stable gene by all algorithms, either alone or among the top-ranked candidates. *gapdh* and *ppib* also showed high overall stability, ranking second and third in the integrated analysis, respectively. In contrast, *actb* was consistently classified as the least stable candidate across the analytical approaches evaluated (Fig. 1; Supplementary Table S2).

The strong performance of *ppia* and *ppib* is consistent with our previous analysis in hamster solid tissues (kidney, liver, and lung), in which cyclophilin-encoding genes also ranked among the most stable candidates [7], consistently outperforming *actb* in both solid organs and blood. A similar pattern has been reported in other animal models, although direct comparisons are limited by differences in species, tissues, and experimental conditions. *Ppia* has shown high stability in blood from pigs following Matrix-M administration and in the spleen of mice infected with *Listeria monocytogenes*, consistent with our findings [21, 22]. In contrast, *gapdh* has shown variable performance, being unsuitable in blood leukocytes from pigs infected with *Actinobacillus pleuropneumoniae* but stable in the liver and kidney of turbot infected with *Edwardsiella tarda* [23, 24]. *actb* appears similarly context-dependent, showing high stability in these turbot tissue but unstable in vaccinated *Senegalese sole* [24, 25]. This discrepancy may, at least in part, reflect differences in cellular composition and immune activation states. Unlike solid tissues, whole blood is a highly dynamic and heterogeneous compartment where shifting leukocyte proportions—such as variations in the neutrophil-to-lymphocyte ratio—can dramatically skew bulk transcriptomic profiles [26]. Furthermore, *actb* encodes a major cytoskeletal structural protein that is rapidly mobilized during immune cell activation, transendothelial migration, and the cytoskeletal rearrangements required for effective pathogen clearance [27]. The systemic inflammatory responses induced by vaccination and active *L. interrogans* infection inevitably trigger significant cytoskeletal remodeling and transcriptional upregulation of *actb* in circulating leukocytes, rendering it highly unsuitable for normalization in this context [27, 28]. In contrast, the high stability of *ppia* across all matrices relies on the broad and constitutive expression of cyclophilin A, a protein whose basal role in cellular homeostasis and protein folding remains uninterrupted by acute leukocyte mobilization [29, 30]. Ultimately, because blood represents a dynamically responding systemic compartment, its reference gene requirements must account for the profound transcriptomic shifts inherent to circulating immune cell activation [31], which frequently disqualify classical, structure-associated genes like *actb*.

#### Condition-specific expression stability analysis

Reference gene stability was also evaluated within each experimental condition to identify the most suitable candidates for specific immunological contexts. Stability rankings varied among groups, indicating that endogenous gene expression was influenced by the type of stimulus applied. Although minor differences were observed among the individual algorithms, the consensus ranking generated using the RefFinder approach implemented in RefSeeker identified distinct optimal reference genes for each condition (Table 1). *rps29* was the most stable gene in the *L. interrogans* bacterin group, whereas *ywhaz* and *aprt* ranked first in the aluminum hydroxide and PBS control groups, respectively. In contrast, *ppia* was the top-ranked candidate in the BCG Pasteur, attenuated *S. Typhimurium*, and active *L. interrogans* infection groups.

In general, RNA-seq-identified genes included in the reference gene panel employed here performed poorly compared with conventional and non-conventional genes, which may be partially explained by the specific biological functions of these novel candidates and their susceptible to modulation during an immune challenge. For instance, leukocyte activation triggers reactive oxygen species (ROS) production, which directly impacts the expression of antioxidant enzymes like *gpx1* [32]. Similarly, the immune-driven activation and proliferation of circulating cells drastically increase protein synthesis demands and immunoregulation, likely destabilizing the expression of ribosomal proteins (*rps16*) [33] and immunophilins (*fkbp1a)* [34]. Therefore, while these RNA-seq-identified genes might present high stability in solid tissues or under steady-state conditions, the highly dynamic and responsive nature of blood during an immune challenge makes them unsuitable as reference genes in this specific context.

The distinct optimal reference genes identified across experimental groups likely reflect the different immune environments induced by each intervention. Leptospiral bacterins and aluminum hydroxide are predominantly associated with humoral responses, whereas BCG and attenuated *S. typhimurium* promote stronger Th1-biased cellular responses, with BCG additionally inducing trained innate immunity [35–38]. Active *L. interrogans* infection represents a markedly different immunological context, characterized by a dysregulated systemic inflammatory response involving pronounced pro- and anti-inflammatory cytokine expression [39, 40]. Because whole blood reflects both the composition and activation state of circulating immune cells, these distinct immune responses may differentially influence the expression stability of candidate reference genes [26, 41]. Accordingly, the differences observed between *rps29* in the bacterin group and *ppia* in the BCG, attenuated *S. Typhimurium*, and infection groups further illustrate the condition-dependent nature of reference gene stability.

Overall, *actb* showed poor stability across most immunized and infected groups, ranking last in several conditions and showing only limited improvement in the PBS control group. *gapdh* and *fkbp1a* also displayed condition-dependent fluctuations, suggesting that their use as universal reference genes in hamster blood should be approached with caution. In contrast, *ppib* ranked among the most stable candidates in several vaccination groups, indicating consistent expression under these conditions, although its performance decreased in samples from infected animals. *ppia*, however, showed high stability in infected animals and maintained favorable rankings across most vaccination settings, emerging as the top-ranked candidate in the BCG Pasteur, attenuated *S. Typhimurium*, and active *L. interrogans* infection groups. Despite the limited sample size employed in this study, the consistency of the stability rankings with our previous study and the relatively low variation in Ct values (Supplementary Figure S1) among biological replicates support the robustness of the findings within the experimental conditions evaluated.

Based on these findings, *ppia* is recommended for studies involving vectored vaccines based on *Salmonella* or BCG, given its high stability in these experimental groups. When interpreting these results, it is important to consider the dynamic nature of systemic immune responses and the sampling strategy used in this study. For vaccinated animals, whole blood was collected 21 days after the booster dose, a time point that likely reflects a more established systemic phase of the immune response. In contrast, infected animals were sampled at the predefined clinical endpoint, representing the acute disease stage. Unlike organ-derived samples, which mainly capture localized events at sites of infection, antigen presentation, or tissue inflammation, blood provides an integrated view of the host response, including circulating immune cells and soluble mediators. This systemic perspective is particularly relevant when comparing infection and vaccination contexts, as the magnitude and distribution of immune mediators may differ substantially between these conditions.

Therefore, although blood represents a practical and less invasive matrix, reference gene stability patterns may differ from those observed in specific tissues or organs. This distinction is particularly relevant in light of our previous validation of reference genes in kidney, liver, and lung samples [7], as blood can provide systemic immunological information while supporting less invasive and potentially longitudinal sampling strategies, reducing the need for terminal tissue collection in some experimental designs [42].

**Table 1:** Stability values and rankings of candidate reference genes across different experimental conditions. Stability was assessed using the comparative ΔCt method, BestKeeper, NormFinder, and geNorm, and the consensus ranking was calculated using the RefFinder approach implemented in the RefSeeker R package.

* Leptospira* Bacterin	delta-Ct		BestKeeper		Normfinder		geNorm		Consensus ranking	
Gene	Avg. STDEV.^a^	Rank	MAD^b^	Rank	Stability	Rank	Avg.M^c^	Rank	Geom. mean value^d^	Rank
*rps29*	1.66	1	3.849	6	0.287	1	0.598	4	2.213	1
*pgk1*	1.696	2	3.426	4	0.287	1	0.839	5	2.515	2
*rps16*	1.817	3	4.304	7	0.591	3	0.349	3	3.708	3
*ywhaz*	1.981	5	4.538	8	1.041	6	0.29	1	3.936	4
*gpx1*	1.983	6	4.561	9	0.998	5	0.29	1	4.054	5
*ppia*	1.947	4	2.928	3	1.146	7	1.193	7	4.924	6
*gapdh*	2.273	9	2.579	1	1.708	9	1.391	9	5.196	7
*ppib*	2.001	7	3.576	5	0.601	4	1.034	6	5.384	8
*fkbp1a*	2.141	8	2.779	2	1.473	8	1.314	8	5.657	9
*aprt*	3.692	10	5.348	10	3.44	10	1.768	10	10	10
*actb*	5.276	11	6.239	11	5.145	11	2.406	11	11	11

BCG	delta-Ct		BestKeeper		Normfinder		geNorm		Consensus Ranking	
Gene	Avg. STDEV.^a^	Rank	MAD^b^	Rank	Stability	Rank	Avg.M^c^	Rank	Geom. mean value^d^	Rank
*ppia*	2.049	1	0.168	1	0.083	1	0.166	1	1	1
*pgk1*	2.073	2	0.17	2	0.228	4	0.166	1	2	2
*ywhaz*	2.174	4	0.263	3	0.211	2	0.407	3	2.913	3
*gapdh*	2.205	5	0.318	4	0.211	2	0.479	4	3.557	4
*rps29*	2.134	3	0.435	5	0.917	5	0.558	5	4.401	5
*rps16*	2.257	6	0.524	6	1.281	7	0.639	6	6.236	6
*gpx1*	2.39	7	0.788	7	1.31	8	0.705	7	7.238	7
*ppib*	2.784	8	1.17	8	2.207	9	0.875	8	8.239	8
*fkbp1a*	3.532	10	1.8	10	1.263	6	1.498	10	8.801	9
*aprt*	3.311	9	1.442	9	2.666	10	1.138	9	9.24	10
*actb*	11.425	11	8.446	11	11.4	11	3.303	11	11	11

Salmonella	delta-Ct		BestKeeper		Normfinder		geNorm		Consensus Ranking	
Gene	Avg. STDEV.^a^	Rank	MAD^b^	Rank	Stability	Rank	Avg.M^c^	Rank	Geom. mean value^d^	Rank
*ppia*	1.495	1	0.409	1	0.065	1	0.13	1	1	1
*ppib*	1.498	2	0.429	2	0.065	1	0.13	1	1.414	2
*rps16*	1.555	3	0.506	4	0.111	3	0.337	5	3.663	3
*pgk1*	1.642	6	0.469	3	0.154	4	0.411	6	4.559	4
*rps29*	1.629	5	0.6	6	0.769	8	0.256	3	5.18	5
*ywhaz*	1.614	4	0.668	7	0.208	5	0.472	7	5.595	6
*gpx1*	1.658	7	0.537	5	0.888	9	0.294	4	5.958	7
*gapdh*	1.687	8	0.798	8	0.481	6	0.554	8	7.445	8
*fkbp1a*	1.856	9	0.969	9	0.693	7	0.642	9	8.452	9
*aprt*	2.669	10	1.449	10	2.26	10	0.873	10	10	10
*actb*	9.443	11	6.959	11	9.425	11	2.432	11	11	11

Al(OH)_3_	delta-Ct		BestKeeper		Normfinder		geNorm		Consensus Ranking	
Gene	Avg. STDEV.^a^	Rank	MAD^b^	Rank	Stability	Rank	Avg.M^c^	Rank	Geom. mean value^d^	Rank
*ywhaz*	0.891	1	0.288	1	0.144	1	0.503	3	1.316	1
*pgk1*	0.937	3	0.468	2	0.361	3	0.452	1	2.06	2
*gapdh*	0.903	2	0.531	5	0.249	2	0.6	4	2.991	3
*ppib*	0.963	4	0.609	6	0.456	4	0.452	1	3.13	4
*rps29*	1	5	0.525	4	0.543	5	0.656	5	4.729	5
*fkbp1a*	1.083	6	0.523	3	0.691	6	0.71	6	5.045	6
*ppia*	1.221	7	0.761	8	0.937	7	0.792	7	7.238	7
*aprt*	1.256	8	0.797	9	1.019	8	0.881	8	8.239	8
*gpx1*	1.584	10	0.76	7	1.421	10	1.072	10	9.147	9
*rps16*	1.442	9	1.031	10	1.272	9	0.962	9	9.24	10
*actb*	1.629	11	1.064	11	1.477	11	1.174	11	11	11

Infection with *L. interrogans*	delta-Ct		BestKeeper		Normfinder		geNorm		Consensus Ranking	
Gene	Avg. STDEV.^a^	Rank	MAD^b^	Rank	Stability	Rank	Avg.M^c^	Rank	Geom. mean value^d^	Rank
*ppia*	2.493	1	1.183	1	0.447	3	0.894	1	1.316	1
*rps29*	2.544	2	1.346	2	0.447	3	0.894	1	1.861	2
*gapdh*	2.605	3	1.642	5	0.273	1	0.959	3	2.59	3
*pgk1*	2.638	4	1.419	3	0.356	2	1.07	4	3.13	4
*ppib*	2.799	5	1.607	4	1.123	5	1.164	5	4.729	5
*ywhaz*	3.161	6	1.931	7	1.827	6	1.75	7	6.481	6
*fkbp1a*	3.191	7	2.079	8	1.849	7	1.497	6	6.964	7
*actb*	4.004	9	1.813	6	3.19	9	2.353	9	8.132	8
*gpx1*	3.935	8	2.215	9	3.124	8	2.081	8	8.239	9
*rps16*	5.94	10	5.388	11	5.563	10	3.003	10	10.241	10
*aprt*	6.285	11	4.244	10	5.896	11	3.6	11	10.741	11

PBS	delta-Ct		BestKeeper		Normfinder		geNorm		Consensus Ranking	
Gene	Avg. STDEV.^a^	Rank	MAD^b^	Rank	Stability	Rank	Avg.M^c^	Rank	Geom. mean value^d^	Rank
*aprt*	2.247	1	1.373	5	0.144	3	0.288	1	1.968	1
*ppib*	2.266	2	1.438	6	0.144	3	0.288	1	2.449	2
*pgk1*	2.296	3	1.638	7	0.103	1	0.329	3	2.817	3
*actb*	2.354	5	1.713	8	0.103	1	0.365	4	3.557	4
*rps16*	2.844	7	0.479	1	1.831	8	1.014	7	4.45	5
*ppia*	2.326	4	1.142	4	0.238	5	0.508	5	4.472	6
*gapdh*	2.717	6	0.767	2	1.553	6	0.832	6	4.559	7
*gpx1*	3.553	9	0.784	3	2.88	9	1.287	8	6.64	8
*fkbp1a*	3.355	8	2.96	9	1.632	7	1.602	9	8.207	9
*rps29*	6.705	10	4.59	10	6.379	10	2.512	10	10	10
*ywhaz*	8.053	11	6.997	11	7.84	11	3.52	11	11	11

^a^Avg. STDEV.: average standard deviation; ^b^MAD: mean absolute deviation; ^c^Avg. M: average expression stability value M; ^d^Geom. mean: geometric mean

### Influence of reference gene selection on target gene quantification

**Fig. 2:**
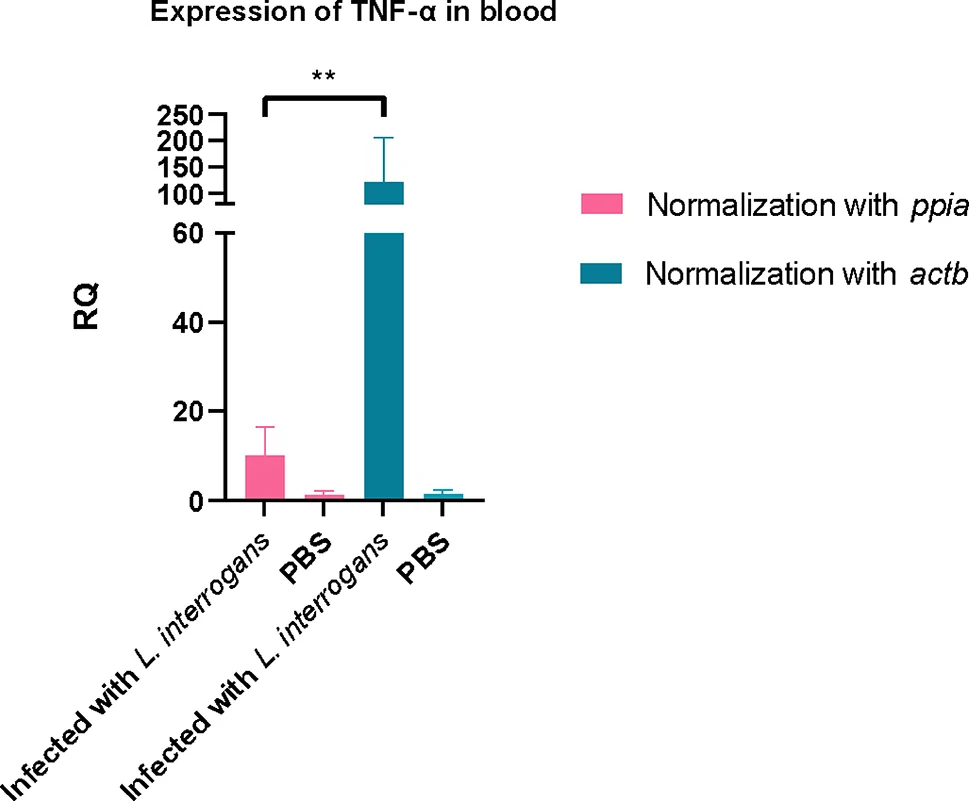
Relative expression of *tnf-α* in blood samples from hamsters infected with *Leptospira interrogans*, normalized using either *ppia* or *actb* as reference genes. For each normalization strategy, the average of all PBS-administered animal samples was used as the calibrator for the ΔΔCt calculation, with the mean relative expression of the PBS group set to 1. Statistical analysis was performed on the resulting normalized expression values to compare the effect of the two normalization strategies on *tnf-α* expression. Double asterisks (**) indicate statistically significant differences (*p* ≤ 0.01). RQ, relative quantification

The relative expression of *tnf-α* was analyzed in blood samples from hamsters infected with *Leptospira interrogans* to determine how reference gene selection affects normalization outcomes. For this analysis, *ppia* and *actb*, identified as the most and least stable genes in the overall RefSeeker ranking, respectively, were used as internal controls. In this analysis, *tnf-α* was used as a target gene because of its central role in inflammatory responses. Normalization with *actb* resulted in an approximately 10-fold overestimation of *tnf-α* expression compared with normalization using *ppia* (Fig. 2), demonstrating the substantial impact of reference gene choice on RT-qPCR data interpretation.

Considering that pairwise variation analysis based on geNorm indicated that three reference genes are sufficient for normalization (Supplementary Figure S3), we also assessed *tnf-α* expression normalized using *ppia* alone versus the combination of the three most stable genes (*ppia*,* ppib*, and *gapdh*). No statistically significant difference was observed when three genes were employed, whereas normalization with the least stable gene, *actb*, resulted in significantly different expression levels (Supplementary Figure S4). We believe that minor differences in V may have limited biological significance due to small sample size and could be attenuated with a larger number of samples. As this assessment is based solely on geNorm, we integrated multiple reference gene stability algorithms using RefFinder to obtain a more robust consensus ranking. This integrated analysis provides additional support for the use of a single reference gene when experimental or practical constraints preclude the combination of three genes.

Although *ppia* performed well in the present study, evidence from other species indicates that its stability may vary according to biological context. For example, *ppia* was reported among the least stable genes in bovine peripheral blood mononuclear cells during haemoparasitic infections [43], reinforcing that reference gene performance is both species- and condition-dependent. In contrast, *actb* showed marked instability across most experimental groups in our analysis, consistent with previous reports showing that classical housekeeping genes can vary substantially across tissues and conditions [44]. Similar context-dependent behavior has also been described for other commonly used reference genes, including *ywhaz* and *gapdh*. These discrepancies may result from biological differences among tissues, species, and experimental stimuli, as well as from methodological differences among stability algorithms.

Together, these findings further support *ppia* as a suitable internal control for hamster blood samples under comparable infection and vaccination conditions and demonstrate that inappropriate reference gene selection can lead to misleading expression profiles and compromise the biological interpretation of RT-qPCR data. Therefore, systematic validation of reference genes remains essential for each experimental model, biological matrix, and treatment condition.

## Conclusion

In conclusion, this study demonstrates that reference gene stability in hamster blood varies according to the immunological condition evaluated, reinforcing the need for experimental validation before RT-qPCR normalization. Among the 11 candidate genes analyzed, *ppia* showed the highest overall stability across infection and vaccination conditions, supporting its use as a reliable reference gene in comparable experimental settings. In contrast, *actb* displayed marked instability in most groups, indicating that its use may compromise the accuracy of gene expression analyses in this model. The evaluation of *tnf-α* expression further confirmed that reference gene selection can substantially affect data interpretation. Together, these findings provide a validated basis for improving the accuracy and reproducibility of RT-qPCR analyses in blood samples from golden Syrian hamsters and support the use of *ppia* as a preferred reference gene for studies involving systemic immune responses.

## Supplementary Information

Below is the link to the electronic supplementary material.

Supplementary Material 1

## Data Availability

No datasets were generated or analysed during the current study.
