# Supplementary material for: Identification and validation of stable reference genes for RT-qPCR analysis in blood samples from golden Syrian hamsters under different immunological conditions
Source: Mol Biol Rep. 2026 Oct 23;53(1):1791. doi: 10.1007/s11033-026-12980-8 (PMC13694556; doi:10.1007/s11033-026-12980-8)
Supplement: Supplementary file 1 [file 11033_2026_12980_MOESM1_ESM.docx]

**Supplementary Table S1.** Primer sequences employed in this study.

| **Gene** | **Genbank accession** | **Primer sequence** | **Amplicon length** | **Efficiency** | **_R_2** |
| --- | --- | --- | --- | --- | --- |

| *actb* | NM_001281595.1 | F: TCTACAACGAGCTGCG | 357bp | 97% | 0.999 |
| --- | --- | --- | --- | --- | --- |
|  |  | R: CAATTTCCCTCTCGGC |  |  |  |
|  |  |  |  |  |  |
| *aprt* | XM_021229013.2 | F:CCTAGACTCCAGGGGCTT  CT | 276bp | 103.5% | 0.999 |
|  |  | R:CTCACACACTCCACCACC  TC |  |  |  |
|  |  |  |  |  |  |
| *fkbp1a* | XM_040746466.1 | F:TCTCGGGACAGAAACAAG  CC | 168bp | 94.0% | 0.999 |
|  |  | R:  ATGTGGTGGGATGATGCCT G |  |  |  |
|  |  |  |  |  |  |
| *gapdh* | DQ403055.1 | F: GGTGCCGAGTATGTTGTGG  A | 270bp | 108% | 0.999 |
|  |  | R: GGCATGGACTGTGGTCATG  A |  |  |  |
|  |  |  |  |  |  |
| *gpx1* | XM_005088272.4 | F:  TTGAGAATGTGGCGTCCCTC | 256bp | 106% | 0.999 |
|  |  | R:GTAAAGAGCGGGTGAGC  CTT |  |  |  |
|  |  |  |  |  |  |
| *pgk1* | XM_040744318.1 | F: GGGTGTGAATCTGCCACAG  A | 115bp | 109% | 0.998 |
|  |  | R:AGCTCCTCCCAAGATAGC CA |  |  |  |
|  |  |  |  |  |  |
| *ppia* | XM_005086775.4 | F:  CGCGTCTCCTTCGAGCTATT | 138bp | 107.5% | 0.999 |
|  |  | R:  ACCCTGGCACATGAATCCT G |  |  |  |
|  |  |  |  |  |  |
| *ppib* | XM_005075522.4 | F:GGCACAGGAGGAAAGAG  CAT | 221bp | 104% | 0.999 |
|  |  | R:  GTGCTCTCCACCTTCCTCAC |  |  |  |
|  |  |  |  |  |  |
| *rps16* | XM_005086220.4 | F:  CTTCAGTCCGTGCAGGTCTT | 90bp | 101% | 0.999 |
|  |  | R:  TCCGTTCACCTTGATGAGCC |  |  |  |

**Supplementary Table S2.** Overall stability values and ranking of candidate reference genes across all blood samples and experimental conditions. The consensus ranking was calculated using the RefFinder approach implemented in the RefSeeker R package.

^a^Avg. STDEV.: average standard deviation; ^b^MAD: mean absolute deviation; ^c^Avg. M: average expression stability value M; ^d^Geom. mean: geometric mean.

| **General** | **delta-Ct** | | **BestKeeper** | | **Normfinder** | | **geNorm** | | **Consensus Ranking** | |
| --- | --- | --- | --- | --- | --- | --- | --- | --- | --- | --- |
| Gene | Avg. STDEV.^a^ | Rank | MAD^b^ | Rank | Stability | Rank | Avg.M^c^ | Rank | Geom. mean value^d^ | Rank |
| *ppia* | 2.477 | 1 | 1.181 | 1 | 0.559 | 1 | 1.021 | 1 | 1.000 | 1 |
| *gapdh* | 2.699 | 3 | 1.239 | 2 | 1.360 | 3 | 1.021 | 1 | 2.060 | 2 |
| *ppib* | 2.581 | 2 | 1.563 | 3 | 0.711 | 2 | 1.301 | 4 | 2.632 | 3 |
| *pgk1* | 2.803 | 4 | 1.597 | 4 | 1.557 | 4 | 1.159 | 3 | 3.722 | 4 |
| *fkbp1a* | 3.052 | 5 | 2.091 | 7 | 1.864 | 5 | 1.650 | 5 | 5.439 | 5 |
| *gpx1* | 3.242 | 6 | 1.769 | 5 | 2.081 | 6 | 2.190 | 7 | 5.958 | 6 |
| *rps29* | 3.419 | 7 | 2.039 | 6 | 2.451 | 7 | 1.967 | 6 | 6.481 | 7 |
| *ywhaz* | 3.567 | 8 | 2.292 | 8 | 2.644 | 8 | 2.399 | 8 | 8.000 | 8 |
| *rps16* | 3.937 | 9 | 2.859 | 9 | 3.154 | 9 | 2.657 | 9 | 9.000 | 9 |
| *aprt* | 4.559 | 10 | 3.254 | 10 | 3.907 | 10 | 3.024 | 10 | 10.000 | 10 |
| *actb* | 5.123 | 11 | 3.659 | 11 | 4.611 | 11 | 3.405 | 11 | 11.000 | 11 |

**Supplementary Figure S1.** Raw Cq value distribution for each gene. Distribution of raw quantification cycle (Cq) values for the 11 candidate reference genes across all evaluated hamster blood samples. Box plots represent the interquartile range (25th and 75th percentiles), with the median indicated by the central horizontal line. Whiskers indicate the range of the data, and colored dots represent individual sample values.

**Supplementary Figure S2.** Melt curve analysis of candidate reference genes. Derivative melt curve plots displaying the change in fluorescence as a function of temperature for the evaluated primer pairs: (A) *ppia,* (B) *gapdh,* (C) *ppib,* (D) *pgk1,* (E) *fkbp1a,* (F) *gpx1,* (G) *rps29,* (H) *ywhaz,* (I) *rps16,* (J) *aprt,* and (K) *actb*.

**Supplementary Figure S3.** Determination of the optimal number of reference genes for accurate RT-qPCR normalization based on pairwise variation (V_n_/V_n+1_) analysis using geNorm.

**Supplementary Figure S4.** Relative expression of *tnf-α* in blood samples from hamsters infected with *Leptospira interrogans*, normalized using either *ppia* individually or *ppia, ppib and gapdh* in combination or *actb* as reference genes. For each normalization strategy, PBS-administered animals were used as the calibrator for the 2^-ΔΔCt^ calculation, with the mean relative expression of the PBS group set to 1. Statistical analysis was performed on the resulting normalized expression values to compare the effect of the two normalization strategies on *tnf-α* expression. Double asterisks (**) indicate statistically significant differences (*p* ≤ 0.01). RQ, relative quantification.
